# Systemic immunologic effects of long-term dupilumab treatment in severe eosinophilic asthma beyond clinical outcomes

**DOI:** 10.3389/falgy.2026.1873964

**Published:** 2026-06-22

**Authors:** Sabina Škrgat, Luka Kunej, Urška Bidovec-Stojkovič, Matevž Harlander, Saša Rink, Peter Korošec, Peter Kopač

**Affiliations:** 1 Department of Pulmonary Diseases and Allergy, University Medical Centre Ljubljana, Ljubljana, Slovenia; 2 Medical Faculty, University of Ljubljana, Ljubljana, Slovenia; 3 University Hospital of Respiratory and Allergic Diseases, Golnik, Slovenia

**Keywords:** aeroallergen-specific IgE, basophils, dupilumab, FeNO, IgE, severe asthma, type 2 inflammation

## Abstract

**Introduction:**

Dupilumab, an IL-4R*α* antagonist that blocks IL-4/IL-13 signalling, is used in severe eosinophilic asthma to improve clinical control and suppress type 2 inflammation.

**Methods:**

We evaluated the clinical and systemic immunologic effects of dupilumab, focusing on type 2 biomarkers, total and aeroallergen-specific IgE, and FcεRI (High affinity IgE receptor) - expressing cells. Thirty-three adults with severe eosinophilic asthma were enrolled and followed up for 12 months in two Slovenian centres. Clinical assessments, spirometry, FeNO (Fractional exhaled nitric oxide), ACT (Asthma Control Test) scores, complete blood counts, total IgE, aeroallergen-specific IgE, and FcεRI expression on basophils were measured at baseline and at 1.5, 3, 6, and 12 months.

**Results:**

Twenty-seven patients completed follow-up and were included in the final analysis. Dupilumab treatment resulted in significant improvement in ACT (*p* = 0.0221), FEV1 (*p* = 0.0139) and reduction in exacerbations requiring OCS (oral corticosteroid) (*p* < 0.0001). FeNO declined by 78 %, total IgE by 83%, and aeroallergen-specific IgE by 65% with consistent reductions across perennial and seasonal allergens. Circulating eosinophils increased transiently, whereas basophils increased by 39%.

**Conclusions:**

Dupilumab demonstrated sustained clinical improvement in patients with severe eosinophilic asthma, accompanied by broad immunologic modulation. Concurrent reductions in FeNO, total IgE, and aeroallergen-specific IgE indicate upstream inhibition of IL-4/IL-13-mediated inflammation, suggesting modulation of the underlying type 2 inflammatory network.

## 1 Introduction

Severe asthma is defined as asthma that remains uncontrolled despite adherence to optimized high-dose inhaled corticosteroid (ICS) and long-acting beta-2 agonist (LABA) therapy, as well as management of contributing factors (1). Although SA affects only about 3%–5% of all patients with asthma (1, 2), it accounts for a disproportionate burden of morbidity, oral corticosteroid (OCS) use, and healthcare costs. Many patients require repeated or chronic OCS therapy, exposing them to significant systemic side effects and reduced quality of life (3)

The majority of patients with severe asthma (approximately 70%–89%) exhibit a type 2 (T2)-high inflammatory phenotype, characterized by elevated biomarkers such as blood eosinophils, fractional exhaled nitric oxide (FeNO), or allergen-specific IgE (4, 5). Among the key mediators of T2 inflammation are interleukin (IL)-4 and IL-13, which induce eosinophilia, mucus hypersecretion, airway remodelling, and B-cell class switching to IgE. Through IgE-mediated activation of mast cells and basophils, these cytokines amplify airway inflammation and bronchoconstriction. IL-4 signalling in particular promotes B-cell class switching toward IgE. IgE, in turn, plays a central role in allergic inflammation by binding to the high-affinity IgE receptor (Fc*ε*RI) on mast cells and basophils (6). Because Fc*ε*RI-mediated activation of basophils represents an important downstream component of IgE-driven inflammation, we additionally evaluated whether dupilumab-associated reductions in total and aeroallergen-specific IgE were accompanied by changes in basophil Fc*ε*RI expression. Upon allergen exposure, this interaction leads to mast cell degranulation and the release of pro-inflammatory mediators such as histamine, leukotrienes, and cytokines—amplifying airway inflammation and bronchoconstriction. Mast cells are present in several airway compartments, including the airway epithelium, smooth muscle, and submucosa, where their activation is associated with more severe disease and poorer symptom control (7, 8).

The introduction of biologic therapies targeting key T2 cytokines has transformed the management of severe asthma. Agents directed against IL-5, IL-4Rα, and IgE—such as mepolizumab, benralizumab, omalizumab, and dupilumab—have shown substantial efficacy in reducing exacerbations, OCS dependence, and improvements in lung function and quality of life (9–12). Dupilumab is a fully human monoclonal antibody that targets the alpha subunit of the interleukin-4 receptor (IL-4Rα), a shared component of the IL-4 and IL-13 receptor complexes. By blocking signal transduction through both pathways, dupilumab suppresses multiple downstream features of T2 inflammation. IL-13 drives airway remodelling via goblet cell hyperplasia, mucus hypersecretion, and subepithelial fibrosis, whereas IL-4 promotes B-cell class switching to IgE and IgG4 (11–14). Through inhibition of these upstream mechanisms, dupilumab not only reduces airway inflammation but also attenuates systemic IgE production. Clinical trials have confirmed that add-on dupilumab therapy reduces OCS use, lowers exacerbation frequency, improves lung function in patients with glucocorticoid-dependent severe asthma and in some patients enables sustained remission of disease (10, 15–17).

In the current era of precision medicine, biomarker-based approaches are increasingly important for guiding biologic therapy in severe asthma. However, longitudinal real-world data on systemic immunologic changes remain limited. In this prospective real-world study, we aimed to evaluate both the clinical and systemic immunologic effects of dupilumab in patients with severe eosinophilic asthma. Specifically, we aimed to characterize changes in type 2 inflammatory biomarkers, including total and aeroallergen-specific IgE, as well as Fc*ε*RI-expressing cells, to better understand the immunologic basis of dupilumab's long-term disease-modifying potential. We hypothesized that dupilumab not only improves clinical control but also induces a broad and measurable downregulation of both total and aeroallergen-specific IgE as part of systemic T2-inflammatory modulation.

## 2 Materials and methods

### 2.1 Study population

Thirty-three adult patients with severe eosinophilic asthma were enrolled in this prospective real-world study conducted between November 2021 and December 2024 at the University Medical Centre Ljubljana and the University Clinic of Respiratory and Allergic Diseases Golnik. All patients met the eligibility criteria for initiating dupilumab therapy, including blood eosinophil count >150 cells/*μ*L and FeNO levels >25 ppb, in accordance with national guidelines (18). Sixteen patients were followed at the pulmonology outpatient clinic in Ljubljana, and 17 at the Golnik outpatient clinic. Fourteen patients had previously received biologic therapy (13 mepolizumab, 1 omalizumab) and were switched to dupilumab because of inadequate clinical response and/or persistent symptomatic chronic rhinosinusitis with nasal polyps despite treatment, immediately after dupilumab became available in Slovenia.

Clinical and immunological assessments were performed at baseline (prior to dupilumab initiation) and at 1.5, 3, 6, and 12 months after starting therapy. Data collected included demographic characteristics (Table 1), pulmonary function tests, FeNO measurements, and Asthma Control Test (ACT) scores and the number of asthma exacerbations. Asthma exacerbations were defined as an event requiring an emergency department visit and/or resulting in hospitalization, or an event requiring treatment with systemic glucocorticoids for ≥3 days; in patients receiving maintenance oral corticosteroid therapy, an exacerbation was defined as the need for at least a two-fold increase in their usual daily oral corticosteroid dose (10). Exacerbation data for the 12 months before dupilumab initiation were collected retrospectively from medical records and patient history obtained during routine clinical assessment. Annualized exacerbation rates were calculated as the number of exacerbations requiring OCS per patient-year during the 12 months before dupilumab initiation and during the first 12 months of treatment. Spirometry was performed using a Vyntus CPX spirometer (CareFusion Germany 234 GmbH) in accordance with the American Thoracic Society criteria. FeNO was measured with the online chemiluminescence analyzer CLD 88 Series (ECOMEDICS, Duernten, Switzerland) according to guidelines from the European Respiratory Society and the American Thoracic Society.

**Table 1:** Patient baseline characteristics.

Characteristic	Value
Prior biologic treatment	
- Biologic-naïve, *n* (%)	**14** (**51.9%)**
- Prior biologic treatment with mepolizumab, *n* (%)	**12** (**44.4%)**
- Prior biologic treatment with omalizumab, *n* (%)	**1** (**3.7%)**
Comorbidities	
- Nasal polyposis, *n* (%)	**20** (**74.0%)**
- Chronic rhinosinusitis, *n* (%)	**11** (**40.7%)**
- Aspirin intolerance syndrome, *n* (%)	**10** (**37.0%)**
- GERD, *n* (%)	**4** (**14.8%)**
- Allergic bronchopulmonary mycosis, *n* (%)	**1** (**3.7%)**
- Atopic dermatitis, *n* (%)	**1** (**3.7%)**
Smoking	
- Past smoker, *n* (%)	**10** (**37%)**
- Never smoked, *n* (%)	**17** (**63%)**
BMI, median (IQR), kg/m^2^	**27** (**23.6–31.5)**
Asthma duration, median (IQR)	**20** (**10-28)**
Maintenance OCS use *n* (%)	**10** (**37.0%)**
Daily OCS dose (prednisone equivalent), median (IQR), mg/day	**5** (**5–20)**
High-dose ICS/LABA use at baseline, *n* (%)	**25** (**92.6%)**
Daily ICS dose, approximate budesonide-equivalent, median (IQR), mcg/day	**1920** (**1440–2400)**

Oral corticosteroid doses were converted to prednisone-equivalent doses. Inhaled corticosteroid doses were standardized to approximate budesonide-equivalent daily doses. ICS/LABA therapy was categorized according to GINA high-dose thresholds. ICS, inhaled corticosteroids; LABA, long-acting β₂-agonists; OCS, oral corticosteroids; GERD, gastroesophageal reflux disease.

Biological treatment with dupilumab was initiated at the Outpatient pulmonology clinic with a loading dose of 600 mg, administered according to the approved dosing guidelines (1). This was followed by maintenance doses of 300 mg every two weeks. Initial doses were given under medical supervision in the clinic, after which patients transitioned to self-administration at home. Clinical follow-up was conducted regularly throughout the treatment period.

The study was approved by the Slovenian Research Ethics Committee (no: 0120-50/2022/3). All study participants gave written informed consent.

### 2.2 Allergy testing and IgE measurement

At enrolment, allergen sensitization was assessed using standard or extended skin prick testing (HAL Allergy, Leiden, Netherlands). A mean wheal diameter of ≥3 mm compared with the negative control was considered indicative of a positive skin test sensitization.

In patients with confirmed atopy, specific IgE levels corresponding to relevant aeroallergens—such as house dust mite, cockroach, cat, dog, grass, and tree pollens, *Candida albicans*, and *Aspergillus fumigatus*—were measured.

Total and allergen-specific IgE concentrations were quantified using the Phadia 250 immunoassay system (Thermo Fisher Scientific, Sweden). A specific IgE concentration ≥0.35 kU/L was defined as positive sensitization.

### 2.3 Peripheral blood collection and cellular immunophenotyping

Peripheral blood samples were collected at each time point (baseline, 1.5, 3, 6, and 12 months) to evaluate haematological and immunological parameters. Complete blood counts were performed, including total leukocyte counts and differential counts of basophils, lymphocytes, monocytes, and granulocytes.

Peripheral blood basophil counts and Fc*ε*RI expression on basophils were assessed by flow cytometry using the DxFLEX Flow Cytometer (Beckman Coulter, USA). Anti-human CD123 PE and HLA-DR PerCP antibodies (both BD Biosciences, USA) were used to identify basophils as CD123 + and HLA-DR– cells. Surface Fc*ε*RI receptors were stained with anti-human Fc*ε*RI FITC (Invitrogen, Thermo Fisher Scientific, USA. The percentage of Fc*ε*RI positive cells was determined individually for each sample based on the corresponding isotype controls (19–24). Flow cytometer MFI was calibrated using Quantum FITC-5 MESF beads (Bangs Laboratories, IN, USA) each time samples were tested. Fc*ε*RI expression is reported as MESF-calibrated receptor density.

### 2.4 Study endpoints

The primary efficacy endpoint was the change in total and allergen-specific IgE concentrations from baseline to 12 months. Key secondary endpoints included improvements in lung function (FEV₁), ACT score, and reduction in exacerbations requiring OCS. Additional endpoints were changes in systemic inflammatory markers and FeNO levels during treatment.

To explore potential biomarkers of treatment response, subgroup analyses were performed based on dupilumab-associated eosinophilia and baseline total IgE levels. Patients were classified as having high (≥1000 cells/µL) or low (<1000 cells/µL) eosinophil counts during treatment, and as having low (<100 kU/L) or high (≥100 kU/L) baseline IgE levels. These groups were compared to assess whether eosinophil dynamics or baseline IgE influenced clinical outcomes.

### 2.5 Statistical analysis

Normality of distribution was assessed using the D'Agostino and Pearson omnibus test. For paired comparisons of normally distributed data the paired t-test was used, while the Wilcoxon signed-rank test was applied for paired comparisons of non-normally distributed data. Two-group comparisons were performed using the Mann–Whitney U test, and categorical variables were analysed using Fisher's exact test.

For paired comparisons between baseline and 12-month measurements, the Wilcoxon signed-rank test was used for non-normally distributed variables, including ACT scores, FeNO, peripheral blood eosinophil counts, Fc*ε*RI expression, total IgE, and aeroallergen-specific IgE levels, while the paired t-test was applied for normally distributed variables, including FEV₁, FVC, Tiffeneau index, and basophil counts.

Two-group comparisons, such as patient stratification according to baseline total IgE levels (<100 vs. ≥100 kU/L) and the magnitude of dupilumab-associated eosinophilia (<1,000 vs. ≥1,000 cells/µL), were evaluated using Mann–Whitney U test.

For the comparison of binomial outcomes, such as high-dose ICS use and occurrence of severe exacerbations in the previous year, Fisher's exact test was used.

Data are presented as median and interquartile range.

Analyses were conducted in GraphPad Prism (GraphPad Software, La Jolla, Calif), version 10.6.1. and R Statistical Computing software version 4.0.2 (R Foundation for Statistical Computing, Vienna, Austria).

Given the exploratory nature of this real-world observational study and the limited sample size, no formal multiple comparison adjustment was applied. *P* values less than 0.05 were considered significant*.*

## 3 Results

### 3.1 Patient population and study flow

**Figure 1:**
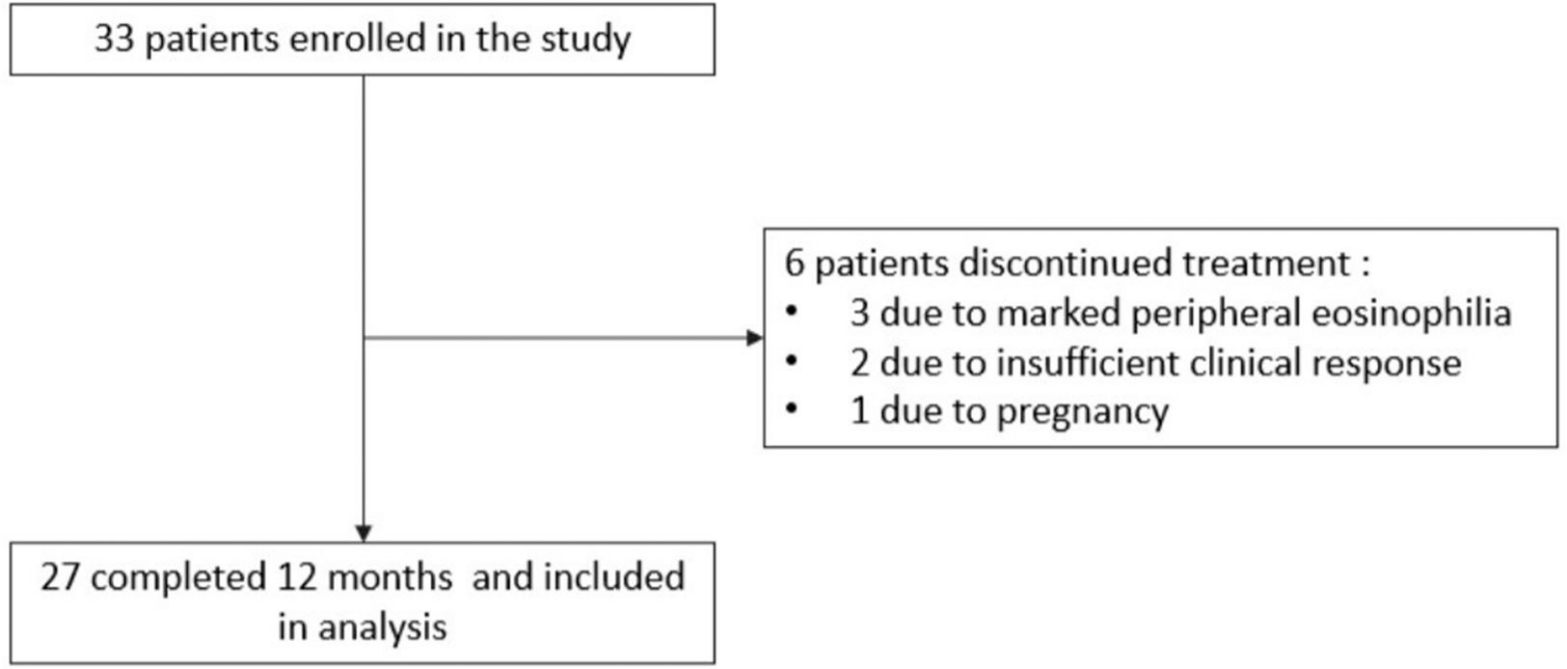
Study flow diagram showing patient enrollment, follow-up, and reasons for discontinuation.

A total of 33 patients were initially enrolled in the study. Of these, 27 completed 12 months of dupilumab therapy and were included in the final analysis. Treatment was discontinued in three patients due to marked increase in blood eosinophil count, in two due to insufficient clinical response, and in one due to pregnancy (Figure 1).

Among the 27 patients included in the final analysis, 14 (51.9%) were biologic-naïve and 13 (48.1%) had previously received biologic therapy, including 12 patients (44.4%) treated with mepolizumab and 1 patient (3.7%) treated with omalizumab.

Clinical and immunological parameters measured at baseline and after 12 months of treatment are summarized in Table 2. Only paired observations were included in the analysis. Aeroallergen-specific IgE, analyses were conducted only in atopic individuals. For aeroallergen-specific IgE, multiple allergen-specific measurements per patient were summarized at the patient level by calculating the median of all sensitized allergen-specific IgE values for each individual at each time point; these patient-level summary values were used for statistical analyses and are reported as median (interquartile range) across the cohort. For paired comparisons between baseline and 12-month measurements, the Wilcoxon signed-rank test was used for non-normally distributed variables, including ACT scores, FeNO, peripheral blood eosinophil counts, total IgE, and aeroallergen-specific IgE levels, while the paired t-test was applied for normally distributed variables, including FEV₁, FVC, Tiffeneau index, and basophil counts. Categorical variables, such as high-dose ICS use and the occurrence of severe exacerbations in the previous year, were analysed using Fisher's exact test.

**Table 2:** Clinical and immunological parameters at baseline and after 12 months of dupilumab treatment.

Clinical and immunological paameters	Baseline	1 year after dupilumab therapy	Change (*Δ*)	95% CI	*p*-value
Number of patients	27	27			
Male patients	12	12			
Female patients	15	15			
Age in years, median (IQR)	55 (48 −65)	56 (49-66)			
Lung function parameters					
FEV_1_ (%), median, (IQR)	67 (54-85)	81 (63–90)	+14	1.78–14.36	***p*** **=** **0.0139**
FEV_1_ (mL), median, (IQR)	2,080 (1,520–2,820)	2,380 (1,930–3,250)	+ 300	56.64–457.20	***p*** **=** **0.0139**
FVC (%), median, (IQR)	87 (78–100)	95 (85–98)	+8	-0.49–7.98	*p* = 0.0809
FVC (mL), median, (IQR)	3,480 (2,800–4,630)	3,750 (2,850–4,700)	+270	-42.41–289.10	*p* = 0.1382
Tiffeneau index, median, (IQR)	64 (52–73)	71 (55–75)	+7	0.59–5.77	***p*** **=** **0.0182**
Asthma control					
ACT, median, (IQR)	17 (12–24)	23 (18–25)	+6	0.00–6.00	***p*** **=** **0.0221**
Use of high-dose ICS, *n* (%)	25 (93)	22 (81)	NA	NA	*p* = 0.4203
Airway inflammation					
FeNO (ppb), median, (IQR)	72 (28–106)	18 (14–23)	-54	-136.00 – -12.00	***p*** **=** **0.0001**
Eosinophilia					
Blood eosinophil count (%), median, (IQR)	3.05 (1.80–6.00)	7.10 (5.30–10.98)	+4.05	0.50–6.30	***p*** **=** **0.0011**
Blood eosinophil count (cells/µL), median, (IQR)	230 (95–463)	540 (328–895)	+310	50.00–680.00	***p*** **=** **0.0016**
Asthma exacerbations					
Number of patients without exacerbation in the last year, *n* (%)	6 (22)	21 (78)	**NA**	**NA**	***p*** **<** **0.0001**
Immunology					
Total IgE (kU/L), median, (IQR)	92 (44–204)	26 (10–60)	-66	-172.70–-33.30	***p*** **<** **0.0001**
Aeroallergen-specific IgE (median per patient) (kU/L), median (IQR)	1.25 (0.14–9.96)	0.22 (0.03–1.16)	-0.85	-6.10–-1.62	***p*** **=** **0.0010**
Basophils (cells/µL), median, (IQR)	24 (18–33)	30 (18–45)	+6	1.29–17.69	***p*** **=** **0.0250**
Fc*ε*RI, median, (IQR)	62,642 (42,346–89,569)	65,950 (39,746–94,743)	+3,308	-19,363–42,859	*p* = 0.2792

Data are shown as median (interquartile range) or number (%), as appropriate. *Δ* represents median paired change. FEV**_1_**, forced expiratory volume in 1 s; FVC, forced vital capacity, ACT, asthma control test; ICS, inhaled corticosteroids; FeNO, ractional exhaled nitric oxide; IQR, interquartile range; NA, not applicable.

The bold values indicate statisticaly significant.

### 3.2 Dupilumab treatment improves lung function, asthma control, and reduces exacerbations

Asthma control, assessed using the ACT, improved significantly during dupilumab therapy. Paired ACT analyses were available for 22 patients, as ACT scores were not recorded at all visits during routine clinical assessment. No imputation was performed; therefore, ACT analyses were restricted to patients with available paired baseline and 12-month values. Among these paired measurements, the median ACT score increased from 17 (12–27) at baseline (indicating poor control) to 23 (18–28) after 12 months (*p* = 0.0221). The proportion of patients with well-controlled asthma (ACT ≥ 20) rose from 33% to 70%.

At study entry, only 22% of patients had experienced no exacerbations requiring OCS in the previous year. The largest subgroup, 33% of patients (*n* = 9), reported 2–5 exacerbations in the past year, while 19% (*n* = 5) had experienced more than five. Overall, the annualized rate of exacerbations requiring OCS decreased from 2.19 exacerbations per patient-year in the year before dupilumab initiation to 0.37 exacerbations per patient-year during the first year of treatment, corresponding to an 83.1% reduction. After 12 months of treatment, 78% of patients reported no exacerbations requiring OCS (*p* < 0.0001), and only a small minority experienced more than one exacerbation during the observation period. In parallel, the proportion of patients receiving high-dose inhaled corticosteroids decreased from 93% to 81% reflecting step-down of ICS therapy in patients who achieved good asthma control, performed as part of routine clinical practice rather than a protocol-based approach.

Treatment with dupilumab also led to a statistically significant improvement in lung function. After 12 months, 74% of patients showed increased absolute and relative FEV₁ values compared with baseline. Median FEV₁ increased from 67% (IQR 54%–85%) to 81% (IQR 63%–90%) of predicted (*p* = 0.0139), corresponding to an increase in absolute FEV₁ from 2,080mL (IQR 1520–2,820 mL) to 2,380 mL (IQR 1,930–3,250 mL; *p* = 0.0139). The Tiffeneau index increased from a median of 64 (IQR 52–73) to 71 (IQR 55–75; *p* = 0.0182). FVC values showed a numerical, but not statistically significant, improvement over the same period.

### 3.3 Dupilumab reduces airway eosinophil inflammation and alters eosinophil dynamics in blood

At baseline, patients had elevated FeNO levels, with a median of 72 ppb (IQR 28–106). During treatment, FeNO declined steadily, reaching a median of 18 ppb (IQR 14–23) after 12 months. This represented a median reduction of 54 ppb and an overall relative decrease of 78% (*p* = 0.0001). Most of the reduction was already evident within the first three months of therapy and was maintained thereafter.

Peripheral blood eosinophil counts showed a different temporal pattern. Following dupilumab initiation, eosinophil counts increased markedly and peaked at three months, with a rise of approximately 268% from baseline. Median eosinophil counts increased from 230 cells/µL (IQR 95–463) at baseline to 630 cells/µL (IQR 370–950) at month 3 (*p* = 0.0019). Thereafter, levels stabilized and declined slightly, reaching a median of 540 cells/µL (IQR 328–895) after 12 months (*p* = 0.0016 vs. baseline). Despite this increase in circulating eosinophils, no associated worsening of asthma control or lung function was observed in the cohort.

### 3.4 Dupilumab significantly reduces total and aeroallergen-specific IgE

**Figure 2:**
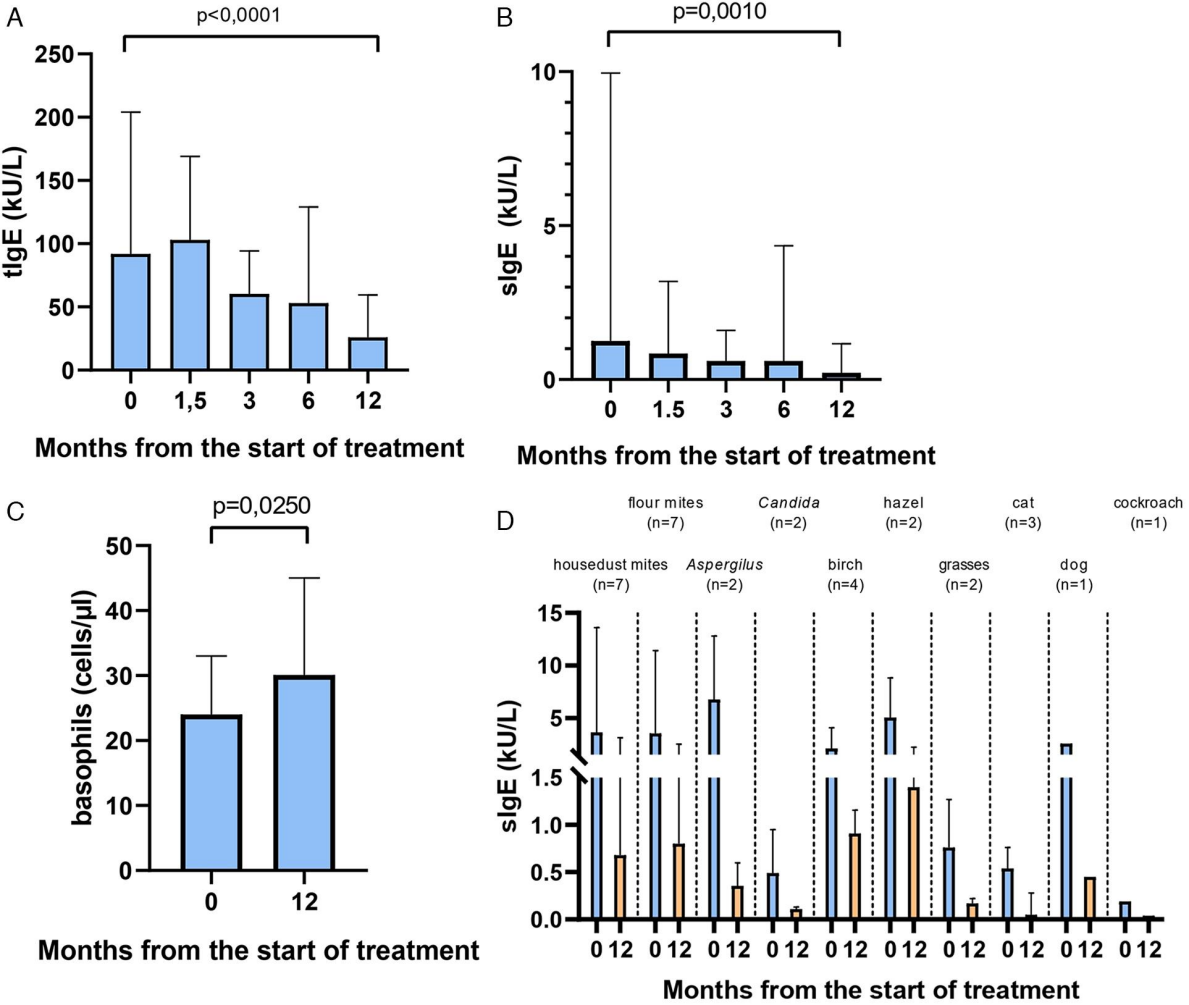
Effects of dupilumab on total IgE, specific IgE, and basophil counts during treatment. **(A)** Total IgE levels measured at baseline and after 1.5, 3, 6, and 12 months of dupilumab therapy show a progressive and significant reduction over time. **(B)** Aeroallergen-specific IgE levels, analysed as patient-level summary measures (median of sensitized allergens per patient), demonstrate a significant decline during treatment. **(C)** Peripheral blood basophil counts at baseline and after 12 months of treatment show a modest but statistically significant increase. **(D)** Aeroallergen-specific IgE levels at baseline (0 months) and after 12 months of dupilumab therapy, stratified by individual allergen sensitization. Reductions in aeroallergen-specific IgE are observed across both perennial and seasonal aeroallergens, including house dust mite, flour mite, birch, cat, grasses, and others.

A total of 27 paired measurements of total IgE were available for analysis. Following the initiation of dupilumab, total IgE levels decreased progressively over the 12-month observation period. Median total IgE decreased from 92 kU/L (IQR 44–204) at baseline to 26 kU/L (IQR 10–60) at 12 months (*p* < 0.0001), corresponding to a relative reduction of approximately 83% (Figure 2A).

Atopy was defined as sensitization to at least one clinically relevant aeroallergen, demonstrated by a positive skin prick test and/or aeroallergen-specific IgE ≥0.35 kU/L. In this subgroup, aeroallergen-specific IgE, levels were monitored according to individual sensitization profiles. We assessed aeroallergen-specific IgE, levels for ten common aeroallergens. The most frequent sensitizations were to house dust mite (*n* = 7) and flour mite (*n* = 7), followed by birch (*n* = 4), cat (*n* = 3), *Aspergillus fumigatus* (*n* = 2), *Candida albicans* (*n* = 2), hazel (*n* = 2), grass (*n* = 2), cockroach (*n* = 1), and dog (*n* = 1) (Figure 2D).

In total, 31 paired aeroallergen-specific IgE, results were available. Aeroallergen-specific IgE values were summarized as the median per patient across all measured allergens. Aeroallergen-specific IgE, levels showed a consistent downward trend during treatment (*p* = 0.0010) (Figure 2B). Median aeroallergen-specific IgE decreased from 1.25 kU/L (IQR0.14–9.96) at baseline to 0.22 kU/L (IQR0.03–1.16) after 12 months, corresponding to a median reduction of −1.03 kU/L. A reduction in aeroallergen-specific IgE was observed across all patients and was not limited to a specific sensitization pattern. Aeroallergen-specific IgE values were also visualized at the level of individual allergens (Figure 2D) to illustrate overall trends, but these data were not used for statistical analysis.

### 3.5 Dupilumab increases circulating basophil counts without affecting Fc*ε*RI expression

An increase in circulating basophil counts was observed during dupilumab treatment. Median basophil counts rose from 24 cells/µL (IQR 18–33) at baseline to 30 cells/µL (IQR 18–45) after 12 months (*p* = 0.0250), This corresponds to a 25% relative increase in the group median. This increase occurred in parallel with the rise in blood eosinophil counts described above (Figure 2C).

By contrast, Fc*ε*RI expression on basophils showed no significant change, remaining stable from 62,642 (IQR 42,346–89,569) at baseline to 65,950 (IQR 39,746–94,743) after 12 months (*p* = 0.2792). A consistent pattern could not be identified; participants demonstrated only small deviations from baseline values.

Over the 12-month period, Fc*ε*RI surface expression remained overall stable despite the marked reductions in total and allergen-specific IgE.

### 3.6 Baseline total IgE and dupilumab-induced eosinophilia do not predict treatment response

Patients were stratified according to baseline total IgE concentrations and the magnitude of dupilumab—associated eosinophilia to facilitate the investigation of potential biomarkers of treatment response.

Subgroup analyses based on baseline total IgE levels (<100 vs. ≥100 kU/L) and the magnitude of dupilumab-associated eosinophilia (<1,000 vs. ≥1,000 cells/µL) were performed as exploratory analyses given the limited sample size.

When stratified by baseline total IgE (<100 kU/L vs. ≥ 100 kU/L), no significant differences were observed in changes in FEV₁ (*p* = 0.4365), FeNO (*p* = 0.1282), peripheral eosinophil counts (*p* = 0.7267), ACT scores (*p* = 0.1443), or basophil counts (*p* = 0.7658) after 12 months of treatment. Both IgE subgroups showed comparable improvements in asthma control and lung function, as well as similar reductions in airway inflammation.

Similarly, when patients were grouped according to the magnitude of eosinophil increase during treatment (≥1,000 cells/µL vs. < 1,000 cells/µL), there were no significant differences in ACT scores (*p* = 0.4945), FEV₁ (*p* = 0.6889), FeNO (*p* = 0.5185), or total IgE levels (*p* = 0.8653). However, patients with a higher increase in eosinophil counts also exhibited a greater increase in basophil counts (*p* = 0.0041). Overall, neither baseline total IgE nor the extent of dupilumab-associated blood eosinophil increase appeared to predict clinical or immunological response to dupilumab.

### 3.7 Treatment discontinuation and safety

During the observation period, dupilumab therapy was discontinued in six out of thirty-three patients.

Treatment was discontinued in three patients due to increased blood eosinophil counts during dupilumab therapy in combination with patient-specific clinical considerations. Peak eosinophil counts in these patients were 1,960, 1,940, and 1,500 cells/µL. In the patient with a peak eosinophil count of 1,500 cells/µL, treatment was discontinued after the occurrence of palpitations and worsening of arterial hypertension. In the patient with a peak eosinophil count of 1,940 cells/µL, dupilumab was discontinued after pulmonary infiltrates were suspected on chest radiography, prompting the introduction of methylprednisolone. Subsequent chest CT did not confirm pulmonary infiltrates, however. This patient was later switched to benralizumab. The patient with a peak eosinophil count of 1,960 cells/µL experienced clinical improvement during dupilumab therapy; however, because of the increase in blood eosinophil counts, treatment was discontinued and the patient was switched to mepolizumab. Overall, these discontinuations were based on the treating physician's clinical judgment during routine care rather than on a predefined protocol-mandated eosinophil threshold. In all three patients, blood eosinophil counts decreased after dupilumab discontinuation.

Two patients discontinued treatment due to insufficient clinical response. One patient discontinued treatment due to pregnancy after nearly one year of therapy, during which her clinical condition had improved.

Adverse events were reported by 12 patients (36.4%). The most reported events were headache (15.2%) and arthralgia or myalgia (15.2%), followed by injection-site reactions (9.1%). No serious adverse events were observed. Safety outcomes are summarized in Table 3.

**Table 3:** Safety outcomes during dupilumab therapy. Patients may have reported more than one adverse event.

Adverse event	n (%)
Patients treated with dupilumab	33
Patients reporting any adverse event	12 (36.4)
Adverse events	
- Headache	5 (15.2)
- Arthralgia/myalgia	5 (15.2)
- Injection-site reaction	3 (9.1)
- Fatigue	2 (6.1)
- Abdominal pain	1 (3.0)
- Ankle swelling (suspected hypersensitivity reaction)	1 (3.0)
- Serious adverse events	0 (0)
Treatment discontinuation	6 (18.2)
- Due to eosinophilia	3 (9.1)
- Due to insufficient response	2 (6.1)
- Due to pregnancy	1 (3.0)

## 4 Discussion

In this prospective real-world study of patients with severe type 2 asthma, dupilumab was associated with substantial improvements in clinical outcomes. Asthma control and lung function improved markedly, and the proportion of patients who remained free from exacerbations requiring OCS increased from 22% at baseline to 78% after 12 months of treatment. These results align with pivotal trials and large registry studies demonstrating the robust efficacy of IL-4R*α* blockade in severe asthma (15, 17, 25).

Airway inflammation was significantly attenuated, as reflected by a nearly 80% reduction in FeNO. Because FeNO is directly regulated by IL-4/IL-13–driven iNOS (inducible nitric oxide synthase) expression, this rapid and sustained decline represents a clear pharmacodynamic signal of effective pathway inhibition (26, 27). In contrast, peripheral blood eosinophil count increased during the first months of treatment, a well-described effect attributed to impaired tissue trafficking rather than enhanced eosinophil production (25, 28). Importantly, this transient eosinophilia was not associated with poorer asthma control or lung function.

An important observation of our study is the parallel reduction in total and aeroallergen-specific IgE. Within the framework of precision medicine, these findings offer real-world evidence on biomarker dynamics that may contribute to the understanding of treatment response and disease modulation. Dupilumab has demonstrated consistent efficacy across several IgE-mediated or type 2–driven diseases, including atopic dermatitis, chronic rhinosinusitis with nasal polyps, asthma, and chronic spontaneous urticaria (29–32). Across these conditions, clinical improvement has been accompanied by a progressive decline in serum total IgE levels, consistent with upstream suppression of IgE synthesis. In asthma, most real-world studies have focused on total IgE and global type 2 biomarkers (10, 15, 17, 33, 34) and data on aeroallergen-specific IgE dynamics remain limited (26, 35, 36). In our study, IL-4R*α* blockade in severe asthma was associated with a sustained reduction in both total and aeroallergen-specific IgE, in line with observations from atopic dermatitis and food allergy (37, 38).

This effect was clearly demonstrable in our cohort: total IgE decreased by 83%, and aeroallergen-specific IgE declined by 65% after 12 months of dupilumab treatment. Reductions were observed across multiple sensitizations, including house dust mite (Der *p* and Der f), cat and dog dander, birch, and grass pollen, indicating a broad and non–allergen-specific suppression of IgE synthesis.

Due to the relatively small sample size, particularly within the atopic subgroup, analyses at the level of individual allergens are limited and should be considered exploratory and hypothesis-generating. Accordingly, no firm conclusions can be drawn regarding differential effects between specific allergens.

Overall, these findings suggest that IL-4R*α* blockade is associated with reductions in both total and aeroallergen-specific IgE.

We also observed that circulating basophils increased by almost 25% after 12 months of dupilumab therapy, consistent with findings in atopic dermatitis and other type 2 diseases (22, 39). This rise might be related to reduced recruitment of basophils into inflamed tissues following IL-4/IL-13 pathway inhibition, resulting in their transient accumulation in the circulation despite overall attenuation of inflammation (22, 23). In contrast, Fc*ε*RI expression remained unchanged, in line with reports that dupilumab—unlike omalizumab—does not directly affect IgE–Fc*ε*RI interactions (24). Despite substantial reductions in total and allergen-specific IgE, these changes were not accompanied by measurable alterations in basophil Fc*ε*RI expression during the 12-month observation period.

Neither baseline total IgE nor the magnitude of dupilumab-associated eosinophilia predicted treatment response. Improvements in ACT, FEV₁, and FeNO were comparable across all subgroups, suggesting that these parameters are not reliable biomarkers for patient selection or treatment monitoring. This observation is consistent with larger cohort studies showing broad dupilumab efficacy across heterogeneous baseline characteristics (15, 17).

This study has several limitations. First, it involved a relatively small cohort drawn from two centres, which may constrain statistical power and limit the generalizability of the findings. Notably, the modest number of atopic patients further reduced statistical power for analyses involving aeroallergen-specific IgE and necessitated the use of patient-level summary measures. Such aggregation may obscure variability in responses to individual allergens.

Second, the observational real-world design lacked a control group, precluding direct causal inference between dupilumab treatment and the observed immunologic changes. According to the study design, several confounders may have influenced the reported results, including natural fluctuations and seasonal variations in IgE levels due to allergen exposure, as well as the potential impact of adjustments in OCS and ICS dosing over the study timeline.

Third, although we comprehensively evaluated clinical outcomes and serum biomarkers, mechanistic assays such as cytokine profiling were not performed. Fourth, aeroallergen-specific IgE was assessed using a limited allergen panel, and the follow-up period was restricted to 12 months; longer observation would clarify whether the observed IgE suppression persists with longer term treatment continuous treatment. Given the number of statistical comparisons performed, the risk of type I error cannot be excluded, and findings—particularly those with borderline statistical significance—should be interpreted with caution.

Finally, basophils were identified using a CD123⁺HLA-DR⁻ gating strategy. No additional exclusion markers were used to specifically exclude plasmacytoid dendritic cells or other CD123⁺ populations. Although such contamination is expected to be limited, it cannot be fully excluded and should be considered when interpreting Fc*ε*RI expression results.

## 5 Conclusions

Dupilumab provides substantial and sustained clinical improvement in severe type 2 asthma outcomes. The parallel reduction in total and aeroallergen-specific IgE, together with FeNO suppression, is consistent with effective IL-4R*α* blockade.

## Data Availability

The raw data supporting the conclusions of this article will be made available by the authors, without undue reservation.
